# Polymicrobial Interactions of Oral Microbiota: a Historical Review and Current Perspective

**DOI:** 10.1128/mbio.00235-22

**Published:** 2022-05-02

**Authors:** Mengshi Zhang, Marvin Whiteley, Gina R. Lewin

**Affiliations:** a School of Biological Sciences and Center for Microbial Dynamics and Infection, Georgia Institute of Technology, Atlanta, Georgia, USA; b Emory-Children’s Cystic Fibrosis Center, Atlanta, Georgia, USA; Nanyang Technological University

**Keywords:** microbiome, microbe-microbe interactions, microbial ecology, oral microbiology

## Abstract

The oral microbiota is enormously diverse, with over 700 microbial species identified across individuals that play a vital role in the health of our mouth and our overall well-being. In addition, as oral diseases such as caries (cavities) and periodontitis (gum disease) are mediated through interspecies microbial interactions, this community serves as an important model system to study the complexity and dynamics of polymicrobial interactions. Here, we review historical and recent progress in our understanding of the oral microbiome, highlighting how oral microbiome research has significantly contributed to our understanding of microbial communities, with broad implications in polymicrobial diseases and across microbial community ecology. Further, we explore innovations and challenges associated with analyzing polymicrobial systems and suggest future directions of study. Finally, we provide a conceptual framework to systematically study microbial interactions within complex communities, not limited to the oral microbiota.

## PERSPECTIVE

The oral diseases caries and periodontitis impact the majority of the adult population in the United States and are two of the most prevalent infections worldwide (1, 2). These diseases are influenced by diverse and dynamic oral microbes and their polymicrobial interactions, such as those mediated by physical attachment or metabolic cues (3–6). Thus, the study of oral diseases has spurned over a century of innovative research at the intersection of pathogenesis and microbial ecology, and the oral microbiota offers an important model to continue to increase our understanding of microbial communities.

## HISTORICAL PERSPECTIVES

### Foundations of oral microbiome studies.

For as long as we have known that microbes exist, we have known about the polymicrobial nature of the oral microbiota. At the discovery of microorganisms in 1683, Antonie van Leeuwenhoek observed a tartar specimen from his tooth using a primitive microscope (7), describing diverse bacterial morphologies that today would be called cocci, spirochetes, and fusobacteria (8). As microbiology research progressed in the late 1800s and early 1900s, scientists worked to discern the role of individual microbes and the collective community as the causative agents of oral diseases (9–11). Although this work was initially restricted to easy-to-culture aerobes and facultative anaerobes such as Streptococcus (11) and *Actinomyces* (12, 13), over time, the challenge of cultivating diverse oral bacteria was overcome by the development of anaerobic cultivation methods for obligate anaerobes and complex media to promote the growth of fastidious bacteria. The successful growth of diverse oral bacteria in the laboratory allowed for the further genotypic and phenotypic characterization of individual species and provided the foundations for polymicrobial studies.

### Polymicrobial biofilm colonization, a spatiotemporal matter.

Research on microbial interactions within the oral microbiota started in 1970 when Gibbons and Nygaard mixed two pure cultures of different species together and observed clumping sedimentation within seconds, initially called interbacterial aggregation (14). While this phenomenon of physical cell-cell interactions, now termed coaggregation, was first described in the oral microbiome, it is prevalent across microbial communities, including those in the human gut, the urogenital tract, and freshwater (15). Coaggregation of oral microbes to each other and adherence to the tooth surface are key to the formation of oral biofilms (plaque) and were comprehensively characterized even before the introduction of the term “biofilm” in 1978 (16).

**FIG 1:**
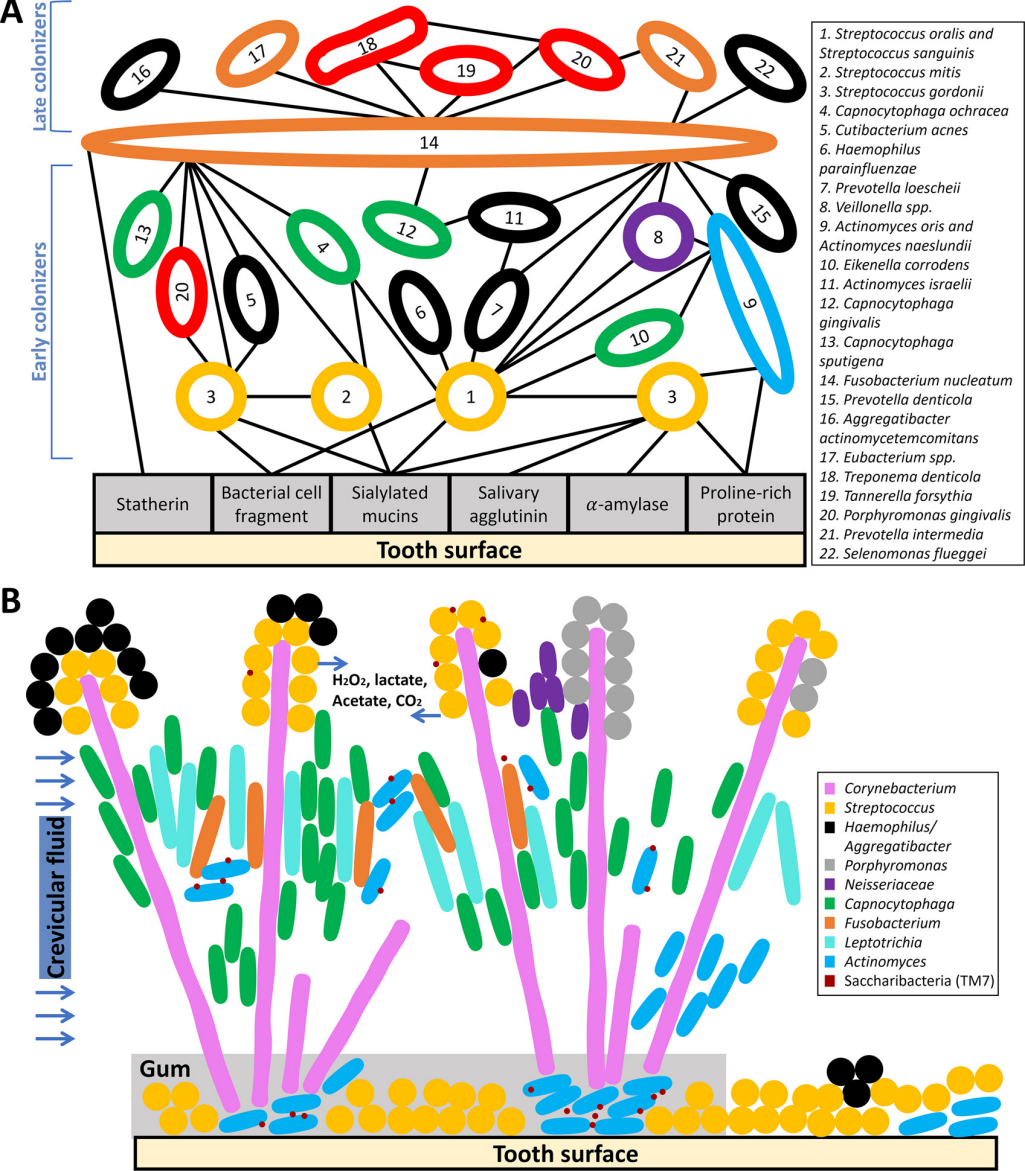
(A) Spatiotemporal model of oral bacterial colonization and pathogenicity. A schematic of the Kolenbrander model of long-term microbial succession from the early stage of biofilm colonization on the tooth surface to the establishment of mature supragingival and subgingival biofilms and, ultimately, to the formation of diseased bacterial communities (17–19). Rods and circles indicate microbial taxa, and lines indicate physical interactions, including binding to the tooth surface or known coaggregation. This schematic integrates over 1,000 coaggregation connections found in the oral cavity, involving microbes that are primarily found both in supragingival and subgingival plaque. The colors of the microbial taxa indicate their corresponding Socransky complex from subgingival plaque, which consists of six categories, yellow, green, blue, purple, orange, and red (25). Specifically, orange and red complexes are more often associated with clinical parameters of gum disease. Species not covered in this Socransky model are colored in black. This schematic highlights that early colonizers and late colonizers are classified into different-colored complexes (57–59) and the proposed importance of F. nucleatum as a bridge species in linking early and late colonizers. Although this model has been highly influential on our understanding of biofilm formation, current work employing advanced microscopy and sequencing techniques continues to refine our understanding of oral biofilm biogeography and development. (B) Supragingival oral biofilm architecture observed using CLASI-FISH, incorporating proposed biochemical gradients and episymbiotic *Saccharibacteria*. In this diagram, the rods and circles indicate microbial taxa, and their locations are based on microscale imaging of supragingival plaque using CLASI-FISH (5, 46). The “hedgehogs” are structured by clusters of *Corynebacterium* filaments that bind to Streptococcus and *Actinomyces* near the base and then expand to the “corncob”-structured perimeter. This spatial patterning also divides the environment into different chemical environments, as shown. The following bacterial genera are colored with their corresponding colored complexes from mature supragingival biofilms: Streptococcus spp. (yellow), *Neisseriaceae* spp. (purple), *Capnocytophaga* spp. (green), *Fusobacterium* spp. (orange), and *Actinomyces* spp. (blue) (27). Other taxa, including *Saccharibacteria*, not included in the Socransky complexes, are shown in other distinct colors. Note that *Porphyromonas* is not included in a colored complex, as *Porphyromonas* here is likely aerotolerant Porphyromonas catoniae and/or Porphyromonas pasteri (46).

To further understand how these interactions influence how bacteria colonize the oral cavity, researchers physically removed mature human oral biofilms and monitored bacterial reappearance on the tooth surface over time. The resulting understanding of the temporal order of appearance of bacterial species combined with possible pairwise coaggregation partnerships enabled the proposal of a spatiotemporal model of oral bacterial colonization summarized by Kolenbrander in 1993 (Fig. 1A) (17–19). After 4 h, the microbes that repopulate cleaned teeth and attach to host surfaces are regarded as “early colonizers” (19). Respectively, the species that colonize at later time points ranging from hours or days to months or years are termed “late colonizers.” While coaggregation is key to tethering these species together, Kolenbrander noticed that early colonizers do not bind to late colonizers and thus proposed the existence of a bridge organism which coaggregates with both early and late colonizers (20, 21). Although rarely found in the first 12 h after professional teeth cleaning, the genus *Fusobacterium* is frequently isolated in both healthy and diseased dental plaques and can bind most genera of early and late colonizers. Thus, *Fusobacterium* is depicted as a coaggregation bridge in the oral microbiome (Fig. 1A) (19).

Together, the ability to bind to the tooth surface and the ability to coaggregate are critical for oral bacterial survival despite constant flow of saliva, regular hygiene practices, variable nutrient supply, and extreme temperature changes. Further, adhesion, coaggregation, and the environmental differences across the surfaces of the mouth result in multiple biogeographical niches in the oral cavity (17, 22). Although biofilm development is important across most ecosystems and, in fact, was first chronicled in aquatic systems in the 1930s, the oral biofilm has provided an accessible and clinically relevant biofilm system (23, 24). Research on the oral biofilm has been fundamental for our understanding of biofilm formation, microbe-microbe interactions, and the relationship between biofilms and pathogenesis (17).

### Polymicrobial biofilms and species associations *in vivo*.

Although the physical interactions in the oral biofilm were widely studied by coaggregation, microbial interaction work specific to plaque from below the gumline (subgingival) was limited until Socransky and his colleagues performed a comprehensive and impactful study in 1998. These authors studied over 13,000 human subgingival biofilm samples from periodontally healthy and diseased patients using checkerboard DNA-DNA hybridization techniques and then grouped 40 culturable bacterial species into six “colored” complexes using cluster analysis and community ordination techniques (Fig. 1) (25). For example, the bacteria in the red complex, Porphyromonas gingivalis, Treponema denticola, and Tannerella forsythia (formerly Bacteroides forsythus), were strongly associated with each other and strongly correlated with periodontal disease. Among the colored complexes, this red complex has attracted significant interest because all three members are putative periodontal pathogens, while other complexes contain both commensals and pathogens (26). The proposal of a red complex contradicted the prevalent idea that disease resulted from the presence of a single pathogen and instead supported the alternative hypothesis that periodontal diseases are associated with communities of bacteria. In addition to identifying the association among species within each complex, Socransky et al.’s study identified the relationship among different complexes. For instance, this work showed that the species in the red complex were rarely found without the presence of orange complex bacteria. This study on subgingival species associations *in vivo* was pioneering and comprehensive, and a similar study was performed using plaque from above the gumline (supragingival) in 2008 (27). While these studies only identified known, culturable bacteria and were constrained by the limitations of correlative studies, they provide important context for future research to study complexes beyond the species level (i.e., strain level or genus level) and to incorporate the role of phenotypic plasticity, including virulence factor production and other responses to host factors (28).

## CURRENT AND FUTURE PERSPECTIVES

### Community composition and function using culture-independent sequencing.

The initial studies on species associations *in vitro* and *in vivo* provided fundamental knowledge for understanding interspecies interactions and biofilm development of oral microbiota. The Socransky et al. study showed the importance of looking beyond interactions among two or a few species. The development of next-generation sequencing techniques and bioinformatic analytic tools allows researchers to study the complexity of diverse oral communities without cultivation, although these studies are challenging due to limited bacterial genetic material in clinical samples and disagreement on the best analysis approaches. The application of 16S rRNA gene amplicon sequencing and metagenomics allows differentiation of bacteria at the species or even strain level and determination of their functional potential.

Work using these techniques has revealed enormous genetic diversity in the human oral cavity: over 700 bacterial species are found in the mouth across individuals, with approximately 200 species per person. Extensive work has resulted in the ability to culture 74% of these microbes (29–31). For instance, the candidate phylum TM7, now *Saccharibacteria*, was discovered 20 years ago using 16S rRNA gene sequence analyses (32), eventually leading to the recent successful cultivation of this microbe with its obligate host bacteria in lab conditions (33, 34). Thus, the initial sequencing analyses led to experimental characterizations and the finding that *Saccharibacteria* downregulates the pathogenicity of its host bacteria and reduces inflammatory bone loss (5).

While the metagenome shows the possible microbial function of the community, it does not reflect the community’s actual activity. A full understanding of oral microbiome dynamics requires a combined picture of microbial composition (the metagenome) and global gene expression (the metatranscriptome). Importantly, the accessibility of the human oral cavity allows for the instantaneous preservation of microbial RNA with temporal and spatial control. Studies using metatranscriptomics on oral specimens have comprehensively shaped our understanding of the oral microbiome. These studies found that although the community composition during periodontitis can vary drastically, the community’s functional activities are conserved (35). This finding suggests that rather than focusing on specific pathogens, we should consider the community as a pathogen (36). Additional support for “the community as pathogen” comes from community-wide metatranscriptomic studies that found organisms traditionally considered commensals transcribe the majority of virulence factors during periodontal diseases (37, 38). Metatranscriptomics can also provide insight into the *in situ* functionality of individual species (39). One striking finding is that a species can have similar relative abundance but different functional activities across different conditions. For example, Fusobacterium nucleatum does not significantly change in relative abundance between healthy and diseased samples, but metatranscriptomics shows that its metabolism is altered (40).

**FIG 2:**
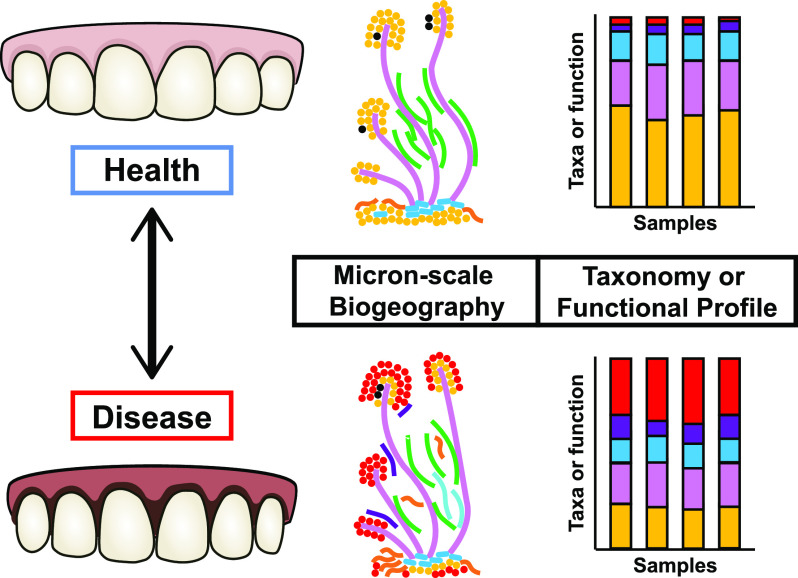
Approaches to studying the dynamics of the oral microbiota during health and disease. Microscopy and sequencing-based community profiling are powerful approaches that can be leveraged for spatiotemporal studies of oral microbial ecology to further understand the relationship of the oral microbiota with health and disease. Confocal scanning microscopy of labeled strains reveals changes in micron-scale biogeography and the corresponding changes in microbial interactions. Community profiling, for instance, using metagenomics and metatranscriptomics, shows changes in the composition and functional activities of samples across space or time. These methods are important in both top-down and bottom-up approaches. For example, top-down approaches could sample the oral biofilm over time after a professional cleaning (60). For bottom-up approaches, emergent spatiotemporal dynamics can be observed using a small number of cells directly removed from oral specimens with micromanipulators or using communities constructed from pure cultures of strains (61).

The oral cavity has become one of the best-described microbial sites in the human body due to its accessibility and clinical relevance; sequencing-driven studies of oral health and disease have broadened our understanding of the community composition and functional activities of the oral microbiota. However, these studies are only starting points, and the driving factors that lead to the progression from healthy to diseased states remain unknown (Fig. 2; Table 1). Longitudinal studies could provide clues about the initial stages of oral diseases and markers of disease progression (38, 41). Finally, there is a further need to simultaneously measure microbial and host gene expression, which could partially explain the development of disease or the shift back to health after therapeutic interventions. These future directions are important for developing therapies to prevent and treat oral infections and for broadly understanding host-associated microbial communities.

**TABLE 1:** Open questions and approaches in oral microbial ecology

Questions	Bottom-up approaches	Top-down approaches
How to build a simplified model system for oral microbiota? What are appropriate metrics for model evaluation?	Experimental model systems can be constructed at the species level (6, 17), genus level (6, 46), or functional level.	Quantitative approaches must be used to benchmark models using the human oral community (39, 42, 56).
What are the similarities and differences in community dynamics between supragingival and subgingival plaque?	Models can illuminate differences in microbial interactions between the two environments. For instance, the Zurich model suggested that the subgingival plaque model could be derived from the supragingival plaque model (62).	Comparative studies using sequencing and microscopy can show intrapatient and interpatient differences between environments.
What are the factors that drive the progression from healthy to diseased states or from diseased to healthy states? How do oral microbes colonize and invade different oral habitats?	Model systems can test the factors that lead to pathogen abundance and the production of virulence factors.	Detailed analyses of longitudinal studies in human patients can further show how communities change over time and which healthy communities become diseased.
How many microenvironments exist in the oral cavity? How do microenvironments impact biodiversity?	Perturbation of laboratory models can test the importance of different environmental factors.	*In situ* measurement of chemical gradients and oxygen levels can indicate different niches. Also, differential abundance of microbes can indicate site specialists with distinct niches (22).

### Microbiogeography.

Despite their utility, metagenomic and metatranscriptomic approaches destroy micron-scale spatial information by homogenizing samples during nucleic acid extraction. Bacteria are micron-sized, and research on oral microbes was the first to show that micron-scale spatial patterning (microbiogeography) can impact disease progression (3, 42). Thus, to understand microbe-microbe and microbe-host interactions, it is additionally important to determine the spatial organization of bacteria with each other and with host factors at scales of microns to hundreds of microns (Fig. 1B; Fig. 2).

In 1972, morphological observation using electron scanning microscopy directly supported that dental plaques form structured biofilms (43). Imaging showed corncob structures composed of central filaments and densely packed layers of cocci attaching to filaments. The advent of fluorescence *in situ* hybridization (FISH) and immunofluorescence allowed researchers to distinguish the identities of microbes in complex oral communities (44). By use of combinatorial labeling and spectral imaging-FISH (CLASI-FISH) (45), the corncob structure was redefined by visualizing nine genera simultaneously in supragingival plaque. The structure was more complex than previously identified: multiple corncobs together formed “hedgehog structures” with aerotolerant taxa on the outside and anaerobic taxa on the inside toward the tooth surface (Fig. 1B) (46). This pioneering study suggested differential oxygen and nutrient usage across the plaque biofilm and provided a spatial framework to incorporate metabolic and ecological factors. Further work quantitatively assessed the proximity of microbes relative to host factors through computing microbial pairwise correlation functions using CLASI-FISH imaging of the tongue dorsum (47). These studies provide important *in situ* benchmarks to experimentally test hypotheses about the spatiotemporal development of the oral biofilm (22, 42).

Applications of CLASI-FISH to visualize microbial structures both in dental plaque and on the tongue show the role of micron-scale interactions in establishing distinctive oral communities. Yet much remains to be learned about how oral microbes organize themselves and respond to gradients of nutrients and oxygen in their preferred environment. Kim et al. provided an example for studying these topics by simultaneously measuring the biofilm architecture, pH microenvironment, and enamel demineralization during the formation of dental caries (6). The authors discovered a rotund-shaped biofilm architecture in intact human dental plaque and quantified the dynamics of microbial community development in lab conditions. This micron-scale patterning resulted in the protection of the oral pathogen Streptococcus mutans by surrounding oral commensals. In another example, to understand how oral microbes disperse and initiate biofilm formation, Simon-Soro et al. combined microscopy with sequencing, showing that most of the microbial biomass in saliva is composed of aggregates containing a mix of both early and late colonizers (48). These diverse aggregates, not single cells, seeded the vast majority of biofilm formation in an *in vitro* model of the tooth surface. This finding provides an alternative to the spatiotemporal model proposed by Kolenbrander and supports the presence of pathogenic microbes early in biofilm formation (18, 49). Together, these innovative studies highlight how research on oral microbes continues to shape our understanding of biofilm formation, microbiogeography, and disease.

There are further opportunities to use imaging approaches such as FISH-based techniques to study disease progression and community dynamics in the oral cavity (Table 1). *In vitro* applications of CLASI-FISH can distinguish up to 120 different species in a single image (50), and high-phylogenetic-resolution microbiome mapping by FISH (HiPR-FISH) can distinguish over 1,000 closely related bacterial strains *in vitro* and has been applied to study oral microbial spatial patterning across longitudinal samples (51). Finally, as metatranscriptomics showed the importance of measuring bacterial function in addition to community composition, there are increasing opportunities to measure bacterial physiology at the micron level (42, 52, 53).

## CONCLUSION

Over a century of work has studied the ecology of oral microbiota, demonstrating how spatiotemporal dynamics lead to oral diseases. Despite this rich history of investigation, there remains much to learn about the emergent properties of oral biofilms and how microbe-microbe and microbe-host interactions influence disease. With diverse microenvironments influenced by dramatic nutrient and temperature changes, regular disturbances (such as hygiene practices), and host interactions, the oral cavity is a valuable model system to study biofilm development, ecological succession, and the eco-evolutionary dynamics that shape an organism’s niche. Given the enormous complexity of oral communities, we suggest that both bottom-up and top-down approaches are important for the further investigation of microbial interactions and the dynamics of oral biofilms (Fig. 2; Table 1). Bottom-up approaches using simplified experimental models provide an easy-access system to evaluate biofilm development and the outcome of clinical interventions. For example, the 10-species “Zurich model” has been used to study topics such as biofilm development, virulence factors, and microbial interactions (54, 55). The advancement of sequencing technologies and microscopy techniques offers further opportunities to design and customize *in vitro* models to match the oral community composition, physiology, and spatial patterning (39, 42, 56), and there are significant opportunities to use these model systems to probe diverse questions (Table 1). In contrast, top-down approaches that directly analyze human patient samples will continue to be essential for understanding the functional and spatial dynamics of oral microbial communities, as discussed in “Current and future perspectives” above.

Research on oral microbes has been critical for our overall understanding of microbial interactions, biofilm development, and spatial patterning and has driven the advancement of technologies for studying microbial communities. Undoubtedly, the oral microbiome provides an accessible and deeply studied system for further exploration of microbial interactions and the role of our microbiota in human health. The future unraveling of the complex dynamics leading to health and disease in the oral cavity will continue to result in new discoveries at the intersection of microbial ecology and pathogenesis.
